# Selective doping of Ni^2+^ in highly transparent glass-ceramics containing nano-spinels ZnGa_2_O_4_ and Zn_1+*x*_Ga_2−2*x*_Ge_*x*_O_4_ for broadband near-infrared fiber amplifiers

**DOI:** 10.1038/s41598-017-01676-6

**Published:** 2017-05-11

**Authors:** Zhigang Gao, Yinyao Liu, Jing Ren, Zaijin Fang, Xiaosong Lu, Elfed Lewis, Gerald Farrell, Jun Yang, Pengfei Wang

**Affiliations:** 1 Key Lab of In-fiber Integrated Optics, Ministry Education of China, Harbin Engineering University, Harbin, 150001 China; 2 Key Lab of Materials for High Power Laser, Shanghai Institute of Optics and Fine Mechanics, CAS, Shanghai, 201800 China; 3 Jiangsu Key Laboratory of Advanced Laser Materials and Devices, School of Physics and Electronic Engineering, Jiangsu Normal University, Xuzhou, Jiangsu China; 4 Optical Fibre Sensors Research Centre, Department of Electronic and Computer Engineering, University of Limerick, Limerick, Ireland; 5 Photonic Research Centre, Dublin Institute of Technology, Kevin Street, Dublin 8, Ireland

## Abstract

Selective doping of Ni^2+^ in octahedral sites provided by nanocrystals embedded in glass-ceramics (GCs) is crucial to the enhancement of broadband near-infrared (NIR) emission. In this work, a NIR emission with a full-width-at-half-maximum (FWHM) of 288 nm is first reported from ZnGa_2_O_4_: Ni^2+^ nano-spinels embedded GCs with excellent transparency. A comparison is made of the NIR luminescence properties of Ni^2+^ doped GCs containing ZnGa_2_O_4_, germanium-substituted ZnGa_2_O_4_ nano-spinels (Zn_1+*x*_Ga_2−2*x*_Ge_*x*_O_4_), and Zn_2_GeO_4_/Li_2_Ge_4_O_9_ composite nanocrystals that are free of Ga^3+^. The results show that ZnGa_2_O_4_: Ni^2+^ GCs exhibit a significantly enhanced NIR emission. The incorporation of the nucleating agent TiO_2_ is favored in terms of the increased luminescence intensity and prolonged lifetime. The possible causes for the enhancement effect are identified from the crystal structure/defects viewpoint. The newly developed GCs incorporate good reproducibility to allow for a tolerance of thermal treatment temperature and hence hold great potential of fiberization via the recently proposed “melt-in-tube” method. They can be considered as promising candidates for broadband fiber amplifiers.

## Introduction

Broadband tunable near-infrared (NIR) light sources are extremely useful for a wide range of applications in optical communications, photochemistry, spectroscopy and pump-probe experiments etc.^1^. The ability of transition metal (TM) ions (e.g., Ti^3+^, V^3+^, Cr^3+^, Cr^4+^, Ni^2+^ etc.) doped bulk crystals and glass-ceramics (GCs) to lase in the broadly tunable NIR (1000~1700 nm) wavelength region have attracted intense attention over the last two decades^2–6^. Because nickel is very stable in its divalent oxidation state (Ni^2+^), Ni^2+^ doped phosphors can be readily synthesized in ordinary lab conditions without strict valence control^7^. However, Ni^2+^ cannot lase in glasses because Ni^2+^ is generally five-fold (trigonal bipyramidally, ^5^Ni^2+^) and/or four-fold (tetrahedrally, ^4^Ni^2+^) coordinated in glassy networks^8^, whereas only six-fold octahedrally coordinated ^6^Ni^2+^ exhibits luminescence in the NIR wavelength range. Even when Ni^2+^ adopts an octahedral coordination in fluoride glasses^9^, the strong electron-phonon coupling of ^6^Ni^2+^ in amorphous materials severely limits the radiative quantum efficiency. These problems can be resolved provided GCs containing nanocrystals that provide octahedral sites for Ni^2+^ can be produced, which is very possible through ingenious composition design and well controlled crystallization^10^. Selective doping of ^6^Ni^2+^ in ZnAl_2_O_4_^11^, LiGa_5_O_8_^12–14^, Ga_2_O_3_^15^ and most recently ZnF_2_ and KZnF_3_^2, 17^ embedded transparent GCs and even glass-ceramic (GC) optical fibers have been produced^16^. Promising results such as the ligand-field driven wavelength tunable and broadband NIR emission of Ni^2+^ have also been observed^5, 17^.

The pursuit of Ni^2+^ doped GCs is being driven continuously by newly invented crystals with excellent luminescence properties, for example, the germanium-substituted ZnGa_2_O_4_ spinel of the general formula Zn_1+*x*_Ga_2−2*x*_Ge_*x*_O_4_ (0 ≤ x ≤ 1) and this has attracted significant attention due to the unprecedented persistent luminescence observed in Zn_1+*x*_Ga_2−2*x*_Ge_*x*_O_4_:Cr^3+^ ^18^. Such nanocrystals can simultaneously provide tetrahedral (occupied by ^4^Zn^2+^ and ^4^Ge^4+^) and octahedral (occupied by ^6^Ga^3+^) sites for Mn^2+^, Co^2+^, Ni^2+^, Cr^3+^ and Mn^4+^ etc., and thus can be used as a multi-functional platform for diverse applications in lighting, display, telecommunication and bio-imaging etc.^19–21^. Additionally, transparent GCs containing Zn_1+*x*_Ga_2−2*x*_Ge_*x*_O_4_ nanocrystals have been recently fabricated^21, 22^. Selective doping of Ni^2+^ in Zn_1+*x*_Ga_2−2*x*_Ge_*x*_O_4_ nanocrystals embedded in GCs has been reported; however, the NIR luminescence properties were not clearly identified in this case^22^. To date the authors of this article are not aware of any study relating to the luminescence properties of ZnGa_2_O_4_: Ni^2+^ GCs, although GCs containing ZnGa_2_O_4_: Cr^3+^ nanocrystals, showing persistent luminescence and temperature sensing properties, have recently been extensively studied^12, 23^.

In the work reported in this article a detailed study has been undertaken and comparison made of the NIR luminescence properties of Ni^2+^ doped GCs containing ZnGa_2_O_4_ and germanium-substituted ZnGa_2_O_4_ (Zn_1+*x*_Ga_2−2*x*_Ge_*x*_O_4_) nano-spinels. To underline the important role of ^6^Ga^3+^, other Ni^2+^ doped GCs containing Zn_2_GeO_4_/Li_2_Ge_4_O_9_ composite nanocrystals that are free of Ga^3+^ were also prepared for comparison. The synthesized ZnGa_2_O_4_: Ni^2+^ GCs are highly reproducible which allows for a tolerance of thermal treatment temperature, and thus are perfectly matched for the recently proposed “melt-in-tube” method. For functional GC fibers cannot be obtained using the conventional “rod-in-tube” method^16, 24–26^, the ‘melt-in-tube method has recently facilitated fabrication of GC fibers doped with Ni^2+^ ^16^, Bi^24^, Cr^3+^ ^25^, or quantum dots^26^ which have exhibited excellent optical quality. The study described in this article is expected not only provide a candidate fiber amplifier material, but also advance understanding of the correlation between the structure of the nanocrystals and NIR luminescence of Ni^2+^, and thus will provide useful guidance for designing novel Ni^2+^ doped GCs with enhanced luminescence properties such as ultra-broadband tunable NIR emission^27^. Moreover, the present work may also advance the understanding of the mechanism underlying the persistence luminescence of Cr^3+^ doped spinels which is currently still open to question^18^.

## Experiments

Three different types of the nominal composition (in mol. %) of Ni^2+^-doped glasses and GCs were prepared using high purity (4N) raw materials of SiO_2_, GeO_2_, Ga_2_O_3_, ZnO, Na_2_CO_3_, K_2_CO_3_, Li_2_O, ZrO_2_, TiO_2_ and NiO.

1. **51SiO**_**2**_ **-18Ga**_**2**_ **O**_**3**_ **-18ZnO-6Na**_**2**_ **O-4ZrO**_**2**_ **-3TiO**_**2**_ **-** ***x*** **NiO** (*x* = 0, 0.15, 0.3, 0.5) was chosen to generate the ZnGa_2_O_4_ nanocrystal, hereafter, this group of glasses and glass-ceramics are denoted as ZGO-*x*PG and ZGO-*x*GC, respectively. Glasses were melted in a platinum crucible at 1600 °C for 2 h, quenched onto a cold brass plate and then annealed at 500 °C for 3 h. GCs were fabricated by heating the annealed glasses at 680 °C for 12 h followed by a further heating at 780~800 °C for 12 h, refer to Tanaka *et al*.^28^;
2. **62GeO**_**2**_ **-20ZnO-10Ga**_**2**_ **O**_**3**_ **-5K**_**2**_ **CO**_**3**_ **-3TiO**_**2**_ **-** ***x*** **NiO** (*x* = 0, 0.15, 0.3 and 0.5) was chosen to generate Zn_1+*x*_Ga_2−2*x*_Ge_*x*_O_4_ nanocrystal, hereafter, this group of glasses and glass-ceramics are denoted as ZGGO-*x*PG and ZGGO-*x*GC, respectively. Glasses were preheated at 850 °C for 30 min and then melted in an alumina crucible at 1400 °C for 30 min. The glasses were then annealed at 500 °C for 3 h. GC was made via a heat-treatment at 650 °C for 2 h, as described in ref. 21;
3. **70GeO**_**2**_ **-15ZnO-15Li**_**2**_ **O-0**.**15NiO** was chosen to generate Zn_2_GeO_4_ and Li_2_Ge_4_O_9_ nanocrystal, hereafter, this group of glasses and glass-ceramics are denoted as ZLGO-0.15PG and ZLGO-0.15GC, respectively. The glasses were melted in a platinum crucible at 1300 °C for 30 min and annealed at 450 °C for 2 h. GCs were fabricated by heating the annealed glasses at 545 °C for 2 h, similar to the process reported in ref. 29.

Transmission spectra were measured using a Perkin-Elmer Lambda 950 UV-VIS spectrophotometer in the spectral range of 200–1800 nm. Refractive indices were measured using an Abbe refractometer AR2008 (KRÜSS, Germany). Photoluminescence (PL) spectra were recorded using a Fluorolog-3-P UV-vis-NIR fluorescence spectrophotometer (JobinYvon, Longjumeau, French). The decay curves were measured using a FLS920 Fluorescence spectrometer (Edinburgh Instruments) from room temperature (300 K) down to liquid helium temperature (10 K). The samples used for the PL measurement were plane-parallel well polished plates with the identical dimension of ~10 × 10 mm^2^ and thickness of 1 mm. The fluorescence was collected in the direction perpendicular to the direction of the pump beam, and the pump light was focused (to a spot of diameter ~4 mm) using a lens and incident at a 45° angle to the normal of the front surface of the sample. In the experiment, both the power of the pump light and the configuration of the light path were kept the same. Because only very thin samples were used for the measurement, reabsorption is not significant and any effects due to this can be omitted in the present study, in accordance with the work of Loiko^30^.

X-ray diffraction (XRD) patterns of all the samples were recorded under the same measurement conditions using an X-ray diffractometer (D/MAX 2550VB/PC, Rigaku Corproation, Japan) with Cu-Kα irradiation. The microstructure of the crystallized glasses was studied using a JEM-2100 high-resolution transmission electron microscope (HRTEM). Raman spectra were measured by RenishawInvia Raman microscope (Renishaw, Gloucestershire, UK) with an excitation wavelength of 515 nm.

## Results and Discussion

**Figure 1:**
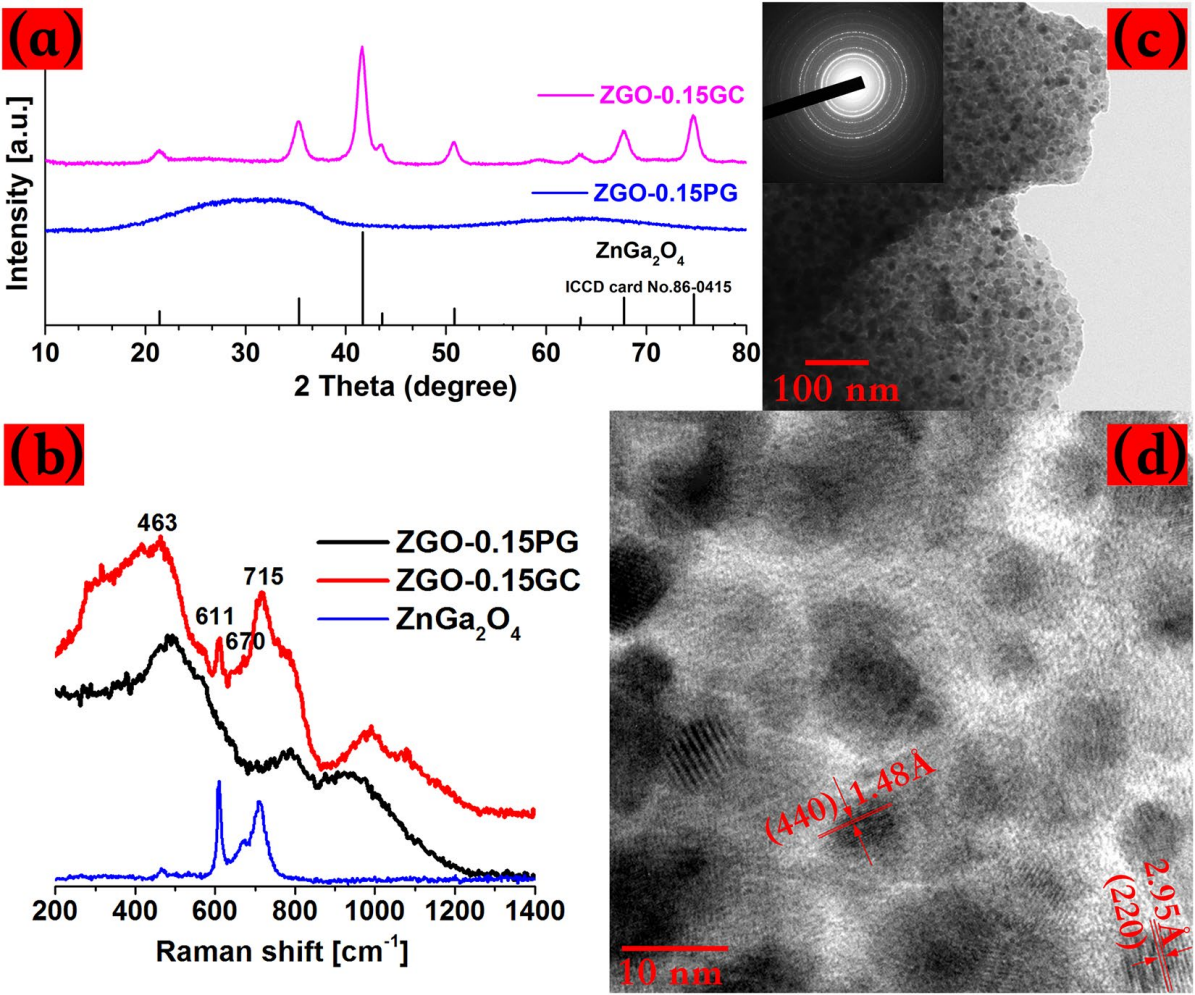
(**a**) XRD patterns of the as-made Ni^2+^ doped glass (ZGO-0.15PG), crystallized glass (ZGO-0.15GC) and standard ZnGa_2_O_4_ crystal (PDF card no. 86–0415); (**b**) Raman spectra of the as-made glass (ZGO-0.15PG), crystallized glass (ZGO-0.15GC) and standard ZnGa_2_O_4_ polycrystals; (**c**) TEM bright-field image of the crystallized glass; (**d**) HRTEM image of the selected area. Inset in (**c**): Selected-area electron diffraction (SAED) pattern.

From the XRD patterns of the crystallized glasses, the precipitation of ZnGa_2_O_4_ (Fig. 1(a)), and Zn_1+*x*_Ga_2−2*x*_Ge_*x*_O_4_ nano-spinels (Fig. S1(a), supporting information), as well as Zn_2_GeO_4_/Li_2_Ge_4_O_9_ composite phases (Fig. S2 in the supporting information) can be discerned in accordance to the literature^21, 28, 29^. The formation of the target nanocrystals were also confirmed from the Raman spectra where the crystallized glasses show sharp scattering peaks well match those of the standard polycrystals (Fig. 1(b)). According to the work of Zhuang *et al*.^21^, it is very difficult to determine unambiguously the exact Zn_1+*x*_Ga_2−2*x*_Ge_*x*_O_4_ phase in GCs, owing to the undistinguishable XRD patterns between the two end-members, ZnGa_2_O_4_ (*x* = 0) and Zn_2_GeO_4_ (*x* = 1). By comparing the Raman spectra of the crystallized glasses with the standard Zn_1+*x*_Ga_2−2*x*_Ge_*x*_O_4_ polycrystals synthesized in our lab by solid-state reaction (for more detail, refer to our previous work^19^), we provide the first direct evidence for the formation of Zn_1+*x*_Ga_2−2*x*_Ge_*x*_O_4_ with x ≥ 0.4 in GCs (Fig. S1(b), supporting information).

The crystallinity (volume fraction of the crystalline phase) of the GCs can be estimated by the ratio of the area under the indexed diffraction peaks to that under the whole XRD patterns^14^. For ZnGa_2_O_4_ and Zn_1+*x*_Ga_2−2*x*_Ge_*x*_O_4_ GCs, the crystallinities are approximately 37% and 32%, respectively, which are close in value to each other. The total molar concentration of ZnO and Ga_2_O_3_ is only 36 mol. % for the ZnGa_2_O_4_ GCs, which is less than the calculated crystallinity. The reason for the discrepancy is not clear and the validity of this result has yet to be confirmed. Here, it should be noted that the presence of nucleating agent such as TiO_2_ in gallium-containing GCs favors the substitution of ^6^Ni^2+^ (ionic radius: 0.69 Å) for ^6^Ga^3+^ (ionic radius: 0.62 Å) via the following substitutional mechanism: Ti^4+^  + Ni^2+^ → 2Ga^3+^, where Ti^4+^ acts as charge compensator^31, 32^. The incorporation of Ti^4+^ in the precipitated nanospinels was confirmed by the TEM-EDS analysis on the selected crystallization area in the ZGO-0.15GC sample (Fig. S3, supporting information). It is possible that a certain degree of inversion may occur in realistic spinels during crystallization, i.e., a fraction of the Ni^2+^ can occupy non-luminescent tetrahedral sites as found in NiAl_2_O_3_ crystals^33, 34^, and hence the selective doping of Ni^2+^ in octahedral sites is highly desirable for enhanced NIR luminescence^33^.

**Figure 2:**
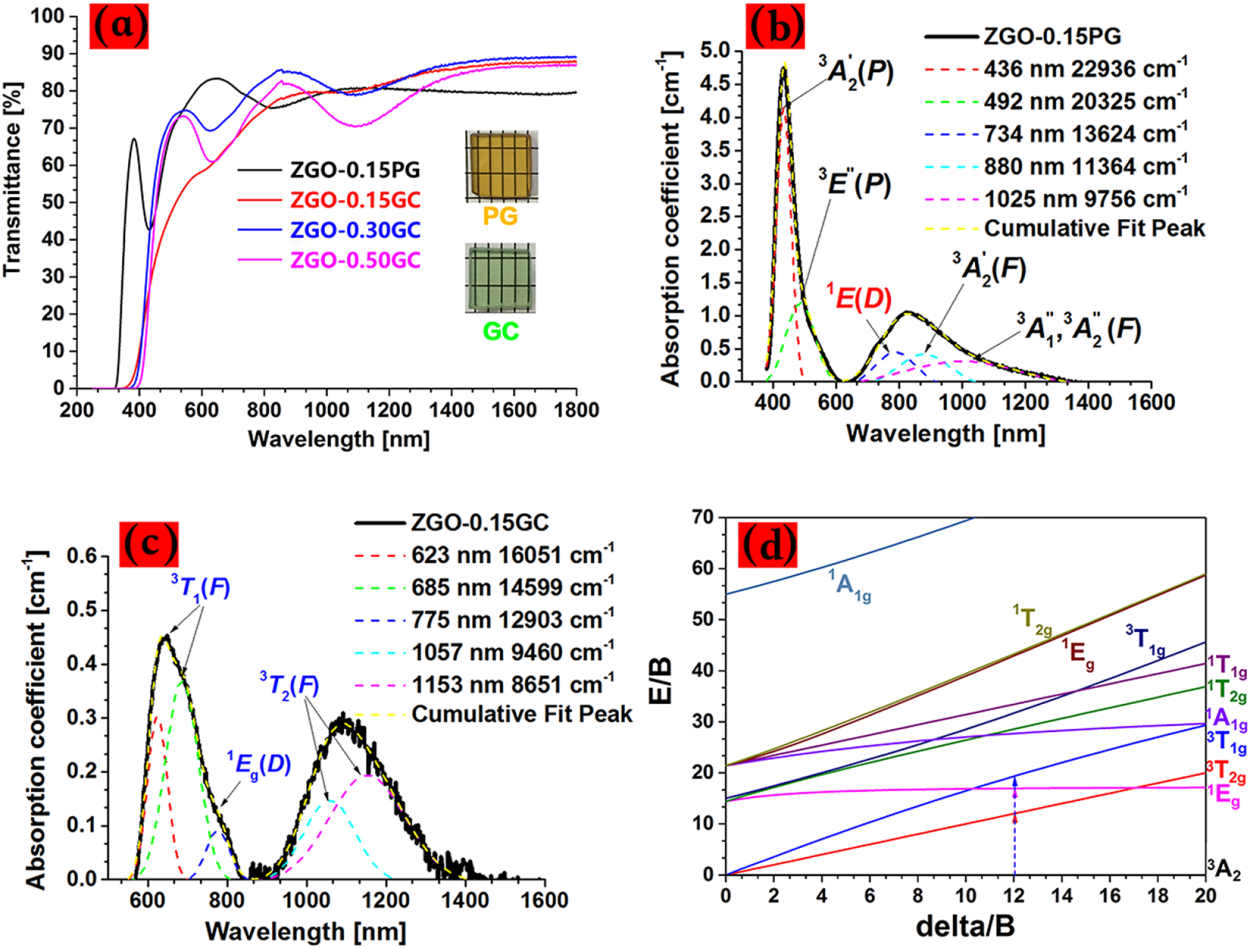
(**a**) Transmission spectra of the ZGO glasses and GCs of varying NiO; Absorption spectra of the ZGO-0.15PG glass (**b**) and ZGO-0.15GC glass ceramic (**c**) obtained after subtracting the background absorption. The absorption bands can be well fitted by Gauss function, and those related to ^6^Ni, ^5^Ni, ^4^Ni are indicated by blue, black and red colors, respectively; (**d**) Tanabe-Sugano (TS) diagram for the octehedrally coordinated Ni^2+^ investigated in the work.

The morphology, distribution and particle sizes of nanocrystals were determined from the HRTEM measurements. The precipitated nanoparticles, approximately 15 nm in diameter, are distributed uniformly in all the GCs (Fig. 1(c) and (d)). The ultra-fine particle size allows these materials to be polished as they are in the glass state and then crystallized without any significant degradation of the surface quality (shown photographically in Fig. 2(a) and Figs S4 and S5, supporting information). The crystallization process was highly reproducible as verified by the fact that GCs show similar performance can be obtained repeatedly under the same experimental condition.

The coordination states of Ni^2+^ can be approximately inferred from the color of the glasses and GCs, for example, blue, brown and yellow-green in the case of ^4^Ni^2+^, ^5^Ni^2+^, and ^6^Ni^2+^ coordination, respectively^8^. The as-made ZGO (Fig. 2(a)) and ZGGO glasses (Fig. S4, supporting information) are light brown in appearance, suggesting ^5^Ni^2+^ and ^4^Ni^2+^ coordination states, whereas the color of the crystallized glasses becomes light green and blue, indicative of ^6^Ni^2+^. Since ^6^Ni^2+^ possesses a larger crystal field stabilization energy (CFSE) value than that of ^5^Ni^2+^, the unstable ^5^Ni^2+^ in glasses tends to transform into ^6^Ni^2+^ during crystallization of the spinel phases. The absorption related to ^6^Ni^2+^ (e.g., around 1160 nm due to the ^3^ *A*_2_(^3^F) → ^3^ *T*_2_(^3^F) transition) in GCs increases with the concentration of NiO, indicative of an efficient partition of Ni^2+^ in ZnGa_2_O_4_ nanocrystals, e.g. more than 90% of Ni^2+^ can be successfully embedded in gallium-containing GCs^7^, whereas it is well known that substitutional doping a large fraction of TM ions into semiconductor nanocrystals is extremely difficult because of the intrinsic self-purification mechanism^35^. The absorption bands can be well fitted to the Tanabe-Sugano (TS) diagram for *d* ^8^ ions (Fig. 2(d)), with the values of Racah parameter (*B*) and crystal field strength (*D*q) equal to 767 cm^−1^ and 917 cm^−1^, respectively.

An inspection of the transmission spectra of the Zn_2_GeO_4_/Li_2_Ge_4_O_9_ GCs (Fig. S5, supporting information) also indicates the presence of ^6^Ni^2+^, which is possible via the substitution of ^6^Ni^2+^ for ^6^Ge^4+^ in Li_2_Ge_4_O_9_ nanocrystals. Meanwhile, since both the valence and ionic radius of ^4^Zn^2+^ (0.60 Å) matches those of ^4^Ni^2+^ (ionic radius: 0.55 Å), the substitution of ^4^Ni^2+^ for ^4^Zn^2+^ in ZnGa_2_O_4_ and Zn_2_GeO_4_ nanocrystals may also occur, similar to the embedding of Ni^2+^ in Zn_2_SiO_4_ crystals^36^. For a detailed analysis and discussion of the absorption spectra, refer to Supporting Information (Fig. S6). The fabricated GCs with transmission larger than 80% demonstrate great potential to be drawn into fibers for use as fiber lasers and amplifiers.

**Figure 3:**
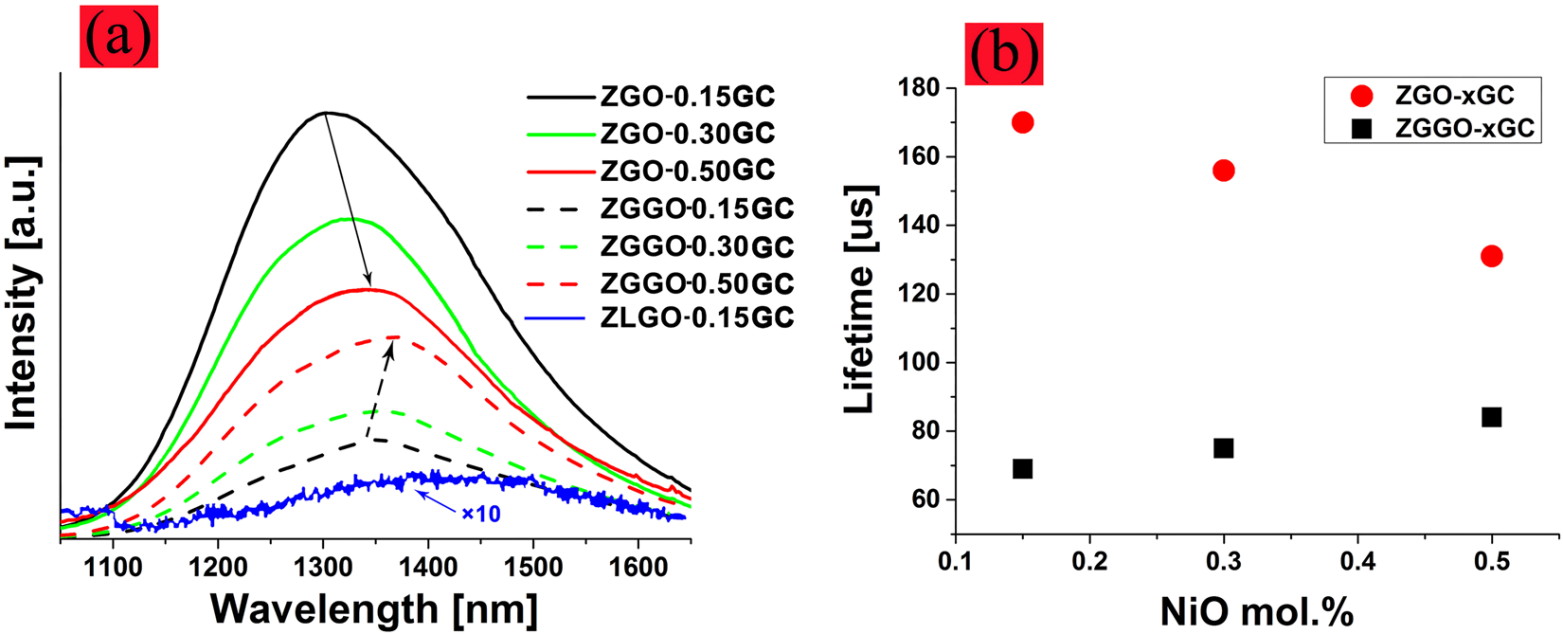
(**a**) Emission spectra of ZnGa_2_O_4_: Ni^2+^ (ZGO-*x*GC, solid lines) and Zn_1+*x*_Ga_2−2*x*_Ge_*x*_O_4_: Ni^2+^ (ZGGO-*x*GC, dashed lines) nanocrystals embedded GCs with varying NiO (*x*, mol. %). Also shown is that of Zn_2_GeO_4_/Li_2_Ge_4_O_9_: Ni^2+^ embedded GCs (ZLGO-0.15GC, dotted line) with the intensity multiplied by an order of ten for the purpose of clarity; (**b**) Lifetime of the NIR emission of the ZGO and ZGGO GCs as a function of NiO.

The use of the nucleant TiO_2_ is very important; it drastically increases both the emission intensity (Fig. S7, supporting information) and lifetime (Fig. S8, supporting information) of the GCs as compared to those free of TiO_2_ but otherwise the GCs containing TiO_2_ were thermally treated under identical conditions. The enhancement effect can be understood based on the substitution mechanism by which Ni^2+^ substitutes for Ga^3+^ favorably as mentioned above. An intense broadband NIR emission (from 1100 to 1700 nm) was recorded from ZnGa_2_O_4_: Ni^2+^ and Zn_1+*x*_Ga_2−2*x*_Ge_*x*_O_4_: Ni^2+^ GCs, but was very weak from Zn_2_GeO_4_/Li_2_Ge_4_O_9_: Ni^2+^ GCs. Both the emission intensity (Fig. 3(a)) and lifetime (Fig. 3(b)) (defined as the time taken for the emission intensity to decay to 1/*e* of its initial value) increase with NiO for the Zn_1+*x*_Ga_2−2*x*_Ge_*x*_O_4_: Ni^2+^ GCs, in contrast to ZnGa_2_O_4_: Ni^2+^ GCs where concentration quenching has already set in at the lowest doping level (~0.15 mol. %). However, in the cases with a fixed NiO, the ZnGa_2_O_4_: Ni^2+^ GCs exhibit stronger NIR emission and longer lifetime than those of Zn_1+*x*_Ga_2−2*x*_Ge_*x*_O_4_: Ni^2+^ GCs, e.g., a five-fold increase in the intensity and a two-fold increase in the lifetime when NiO was 0.15 mol. %. For the Zn_1+*x*_Ga_2−2*x*_Ge_*x*_O_4_: Ni^2+^ GCs, the emission intensity appears not to saturate at 0.5 mol. %. GCs have also been fabricated doped with 0.7 mol. % NiO. However, the samples suffer from significant devitrification due to NiO-assisted growth of large sized crystals commonly found in glasses heavily doped with NiO^7^. As a result, the 0.7 mol. % doped GCs become opaque and this restricts their use for optical applications, and hence does not warrant further study.

The differences in the crystal structures between the ZGO and ZGGO GCs may account for the contrast in the luminescent property. The Zn_1+*x*_Ga_2−2*x*_Ge_*x*_O_4_ crystals were assumed to be a solid solution between the normal ZnGa_2_O_4_ and inverted Zn_2_GeO_4_ spinel structures^19, 37^. Pure Zn_1+*x*_Ga_2−2*x*_Ge_*x*_O_4_ spinels can be synthesized for *x* ranging from 0 to 0.5^19^. Our recent study of Mn doped Zn_1+*x*_Ga_2−2*x*_Ge_*x*_O_4_ phosphors shows that the substitution of Ge^4+^ for octahedrally coordinated ^6^Ga^3+^ helps to separate Mn^4+^ which also substitutes for ^6^Ga^3+^, thus resulting in an enhanced emission of Mn^4+^ ^38^. It is assumed that a similar separating effect exists for Ni^2+^ as well, i.e., ^6^Ni^2+^ ions are well separated in the ZGGO GCs, and thus the concentration quenching is postponed. As more Ni^2+^ ions diffuse from the surface to the inside of the nanocrystals and/or are shielded by the nanocrystals from the outside high-phonon energy environment, the non-radiative relaxation rate is reduced, and the lifetime increases accordingly. However, in the case of ZnGa_2_O_4_ GCs, Ni^2+^ substituting for ^6^Ga^3+^ is not well separated because there is no “separating agent” akin to Ni^2+^ doped Ga_2_O_3_ GCs where concentration quenching already starts at 0.10 mol. % low content of Ni^2+^ ^7^, and this accounts for the observed decreasing lifetime in the ZGO GCs.

Previous studies have shown that the Cr^3+^-doped and germanium-substituted compounds (Zn_1+*x*_Ga_2−2*x*_Ge_*x*_O_4_, *x* ≤ 0.5) exhibit much brighter and longer persistence luminescence than pure Cr^3+^-doped ZnGa_2_O_4_ spinels^37^. The 2Ga^3+^ → Ge^4+^ + Zn^2+^ substitution induces an inversion increase in the spinel structure, that is, an increased amount of Ga^3+^ now occupies the tetrahedral ^4^Zn^2+^ sites, forming the so-called anti-site defects $({{\rm{Ga}}}_{{\rm{Zn}}}^{\bullet })$. According to ref. 37, the enhancement of Cr^3+^ emission relies on the formation of anti-site defects, however, the presence of such defects definitely has an adverse effect on the luminescence of Ni^2+^ because of the reduced proportion of ^6^Ga^3+^ sites. On the other hand, it is possible that the substitution of Ge^4+^ for ^6^Ga^3+^ may generate octahedrally coordinated ^6^Ge^4+^, which may in turn be substituted by Ni^2+^. However, considering the fact that only weak NIR emission was observed from the Zn_2_GeO_4_/Li_2_Ge_4_O_9_: Ni^2+^ GCs, and the large mismatch in valence and ionic radii between ^6^Ni^2+^ and ^6^Ge^4+^ (ionic radius: 0.53 Å), the substitution of ^6^Ni^2+^ for ^6^Ge^4+^ is severely limited, akin to the partition of Ni^2+^ in K_2_SiF_6_ nanocrystals embedded GCs^17^. Moreover, no NIR emission related to the tetrahedrally coordinated ^4^Ni^2+^, e.g., Ni^2+^ doped Zn_2_SiO_4_ or Zn_2_GeO_4_ crystals, has been recorded even at cryogenic temperatures^36^. All these effects account for the observed weaker emission intensity of the ZGGO GCs than that of the ZGO GCs.

**Figure 4:**
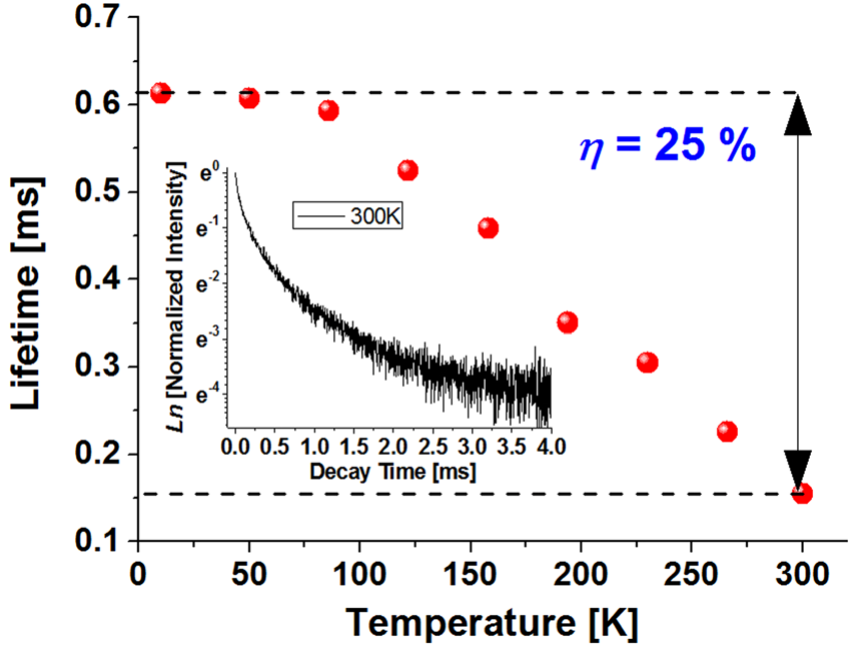
Temperature dependence of the NIR emission lifetime of Ni^2+^ doped ZGO GCs. Inset: the decay curve of the NIR emission of Ni^2+^ at the room temperature, those measured at other temperatures have a similar profile and thus are not shown.

The ZnGa_2_O_4_: Ni^2+^ GCs, were selected for further study of internal fluorescence quantum efficiency (*η*) due to the stronger NIR emission and longer lifetime of Ni^2+^. Figure 4 shows the NIR emission lifetime of Ni^2+^ as a function of temperature from room temperature (300 K) down to liquid helium temperature (10 K). The sudden drop in lifetime at around 100 K indicates the occurrence of phonon-assisted non-radiative relaxation^39^. As shown in the inset, the decay curve has a strong non-exponential characteristic, implying multiple site effects of Ni^2+^ and non-radiative multipolar interactions among Ni^2+^. The value of *η* can be calculated as *η *=* τ*_300K_/*τ*_0K_, where *τ*_300K_ (~0.17 ms) and *τ*_0K_ (~0.62 ms, obtained by linear extrapolation to 0 K) are the lifetimes at the room and absolute zero temperatures, respectively. It is about 25% for the ZnGa_2_O_4_: Ni^2+^ GCs, which is less than that of ZnAl_2_O_4_: Ni^2+^ (~55%)^40^, LiGa_5_O_8_: Ni^2+^(~60%)^12^ and BaAl_2_Ti_6_O_16_: Ni^2+^ (~65%)^39^ GCs. However, it is comparable to that of Ga_2_S_3_: Cr^4+^ chalcogenide GCs (~25%)^4^ and even larger than that of pure Ni^2+^ doped ZnGa_2_O_4_ crystals (~18%)^41^.

The stimulated emission cross section (σ_e_) was calculated using the McCumber formula^4^ and was found to be 0.52 × 10^−20^ cm^2^. The product of σ_e_ and *τ*_300K_ (proportional to the amplification gain and inversely proportional to the laser oscillation threshold) taken as a figure of merit (FOM) for the ZGO-0.15GC sample is 1.23 × 10^−24^ cm^2^·s if the 1/*e* lifetime is used for the calculation, and is about 3.79 × 10^−24^ cm^2^·s if the average lifetime $\,{\tau }_{{\rm{m}}}=\int tI(t){\rm{d}}t/\,\int I(t){\rm{d}}t$ is used for the calculation, which is comparable to that of ZnAl_2_O_4_: Ni^2+^ (~3.1 × 10^−24^ cm^2^·s)^40^, LiGa_5_O_8_: Ni^2+^ (~3.7 × 10^−24^ cm^2^·s)^12^ and BaAl_2_Ti_6_O_16_: Ni^2+^ (~3.3 × 10^−24^ cm^2^·s)^39^ GCs, and much larger than Ga_2_S_3_: Cr^4+^ chalcogenide (ChG) GCs (~0.62 × 10^−24^ cm^2^·s) which are known for the difficulty of preparation^4^. Light amplification at similar *O*-band wavelengths can be also achieved for Pr^3+^ or Dy^3+^ doped fluoride and ChG glasses of very low phonon energy. In comparison, the FOM of the ZGO-0.15GC is less than that of Pr^3+^ doped Ge-Ga-S ChG glass (4.79 × 10^−24^ cm^2^·s)^42^, however, it is much larger than in the case of Pr^3+^ doped ZBLAN (0.38 × 10^−24^ cm^2^·s) and Dy^3+^ doped Ge-Ga-S ChG glasses (1.4 × 10^−24^ cm^2^·s)^43, 44^. Moreover, the present GCs are superior to rare-earth doped glasses in terms of the availability of a broad tuning range of wavelength. A comparison of the luminescent properties (*λ*_peak_, peak emission wavelength, *τ*_298K_, decay lifetime at the room temperature, and FOM) and crystal field parameters (*D*q and *B*) of Ni^2+^ in GCs containing different spinels is shown in Table 1. The magnitude of crystal field strength *D*q is a measure of the interaction of the 3*d*-electrons with the rest of the lattice, and the main contribution arises from the nearest neighbors. Although Ni^2+^ substitutes for Ga^3+^ in both ZGO and ZGGO GCs, the *D*q value of the latter is slightly less than that of the former GCs, which, according to the ligand field theory, is due to the distortion of ligands inducing a weakening effect on the crystal field strength of the central ion^45^.

**Table 1:** Comparison of luminescent properties and crystal field parameters of GCs containing different nanospinels. Rare earth (Pr^3+^ or Dy^3+^) doped glasses emitting at similar *O*-band wavelengths are also listed for comparison.

	ZnGa_2_O_4_	Zn_1+*x*_Ga_2−2*x*_Ge_*x*_O_4_	(Ga_2_O_3_)_3_(GeO_2_)_2_	LiGa_5_O_8_	*β*-Ga_2_O_3_	ZnAl_2_O_4_	Pr^3+^	Dy^3+^
^3^ *T*_2g_(^3^F) (cm^−1^)	9174	9091	9708	9483	9891	9066		
^1^ *E*_g_(^1^ *D*) (cm^−1^)	12903	12903	13737	12987	13158	14124		
^3^ *T*_1g_(^3^F) (cm^−1^)	15504	15149	16189	15949	16502	14517		
*D*q (cm^−1^)	917	909	971	948	989	907		
*B* (cm^−1^)	767	712	887	895	892	940		
*λ*_peak_ (nm)	1320	1350	1300	1300	1200	1350	1344	1340
*τ*_298K_ (μs)	170^†^/525^‡^	70^†^/258^‡^	254^‡^	583^‡^	665^‡^	240^‡^	360^†^	38^†^
FOM × 10^−24^ cm^2^·s	1.23^†^/3.8^‡^	/	/	3.7^‡^	/	3.1^‡^	4.79^†^	1.4^†^
Ref.	this work	this work	5	12	45	31, 40	42	43–44

^†^1/*e* lifetime and ^‡^average lifetime.

It is important to stress that the synthesized ZnGa_2_O_4_: Ni^2+^ GCs are highly reproducible to allow for a fluctuation in the thermal treatment temperature, for example, transparent GCs with the broadband near NIR emission can be obtained at a crystallization temperature ranging from 750 to 800 °C (Fig. S9 in the supporting information). This is a very important advantage for the “melt-in-tube” method, for which the core fiber is covered with the SiO_2_ cladding, and the heat transfer process during the heat treatment can be different from that of the glass sample. Because of different thermal treatment temperature, higher for the “melt-in-tube” method, GCs with required luminescent properties and transparency should be obtained in a temperature range as broad as possible. In this respect, the studied ZGO GCs are perfectly matched to the “melt-in-tube” method, which will be the subject of our next study to succeed in making them into fibers.

## Conclusion

The selective doping of Ni^2+^ in ZnGa_2_O_4_ and Zn_1+*x*_Ga_2−2*x*_Ge_*x*_O_4_ nano-spinels via the controlled crystallization results in a broadband NIR emission. The use of nucleating agents such as TiO_2_ promotes occupation of the octahedral Ga^3+^ sites by Ni^2+^ and leads to enhanced luminescence and prolonged lifetime, whereas the partition of Ge^4+^ in ZnGa_2_O_4_ spinels leads to a reduced NIR emission, which is assumed to be related to the formation of anti-site defects. The large mismatch of valence and ionic radii between ^6^Ni^2+^ and ^6^Ge^4+^ considerably limits the substitution of ^6^Ni^2+^ for ^6^Ge^4+^, which also partly accounts for the comparatively weaker NIR emission from the Zn_1+*x*_Ga_2−2*x*_Ge_*x*_O_4_: Ni^2+^ GCs. The stronger NIR emission, excellent optical quality and reproducibility, as well as a tolerance for thermal treatment temperature make ZnGa_2_O_4_: Ni^2+^ nano-spinels embedded GCs highly promising candidates for broadband fiber amplifiers. Future work will focus on fabricating GC fibers by the “melt-in-tube” method.

## Electronic supplementary material

Supporting Information
