# Supplementary material for: Selective doping of Ni2+ in highly transparent glass-ceramics containing nano-spinels ZnGa2O4 and Zn1+xGa2−2xGexO4 for broadband near-infrared fiber amplifiers
Source: Sci Rep. 2017 May 11;7:1783. doi: 10.1038/s41598-017-01676-6 (PMC5431766; doi:10.1038/s41598-017-01676-6)
Supplement: Supplementary file 1 — Supporting Information [file 41598_2017_1676_MOESM1_ESM.doc]

Supporting information for

**Selective doping of Ni^2+^ in highly transparent glass-ceramics containing nano-spinels ZnGa_2_O_4_ and Zn_1+_*_x_*Ga_2-2_*_x_*Ge*_x_*O_4_ for broadband near-infrared fiber amplifiers**

**Zhigang Gao ^1^, Yinyao Liu ^2^, Jing Ren ^1, 3,*^, Zaijin Fang ^1^, Xiaosong** **Lu ^1^, Elfed Lewis ^4^, Gerald Farrell ^5^, Jun Yang ^1^ and Pengfei Wang ^1,5,*^**

^1^Key Lab of In-fiber Integrated Optics, Ministry Education of China, Harbin Engineering University, Harbin 150001, China

^2^Key Lab of Materials for High Power Laser, Shanghai Institute of Optics and Fine Mechanics, CAS, Shanghai 201800, China

^3^Jiangsu Key Laboratory of Advanced Laser Materials and Devices, School of Physics and Electronic Engineering, Jiangsu Normal University, Xuzhou, Jiangsu, China

^4^Optical Fibre Sensors Research Centre, Department of Electronic and Computer Engineering, University of Limerick, Limerick, Ireland

^5^Photonic Research Centre, Dublin Institute of Technology, Kevin Street, Dublin 8, Ireland

Corresponding author: ren.jing@hrbeu.edu.cn (J. Ren), [pwang@hrbeu.edu.cn](mailto:pwang@hrbeu.edu.cn) (P. Wang)

Formation of the pure Zn_1+_*_x_*Ga_2-2_*_x_*Ge*_x_*O_4_ phase can be inferred from the Raman spectrum of the ZGGO-0.15GC sample (Fig. S1(b)). For the GC sample, Raman features at 610, 722 and 801 cm^-1^ can be observed. According to our previous study of the Zn_1+_*_x_*Ga_2-2_*_x_*Ge*_x_*O_4_ polycrystals [[[1]](#endnote-2)], the vibrational bands at 610 and 722 cm^-1^ correspond to the [Ga-O-Zn] modes and the vibrational band at 801 cm^-1^ is due to the [GeO4] mode. The Raman features of the crystallized glass agree favorably with those of the Zn_1+_*_x_*Ga_2-2_*_x_*Ge*_x_*O_4_ polycrystals for *x* ranging from 0.4 to 0.75. However, the exact value of *x* still cannot be accurately determined because the features we referred to all exhibit in the Raman spectra of the Zn_1+_*_x_*Ga_2-2_*_x_*Ge*_x_*O_4_ compounds for *0.4≤x≤ 0.75.* Therefore, the only conclusion we can draw is that the precipitated nanocrystals are possibly the Zn_1+_*_x_*Ga_2-2_*_x_*Ge*_x_*O_4_ phase with *x* ≥ 0.4.


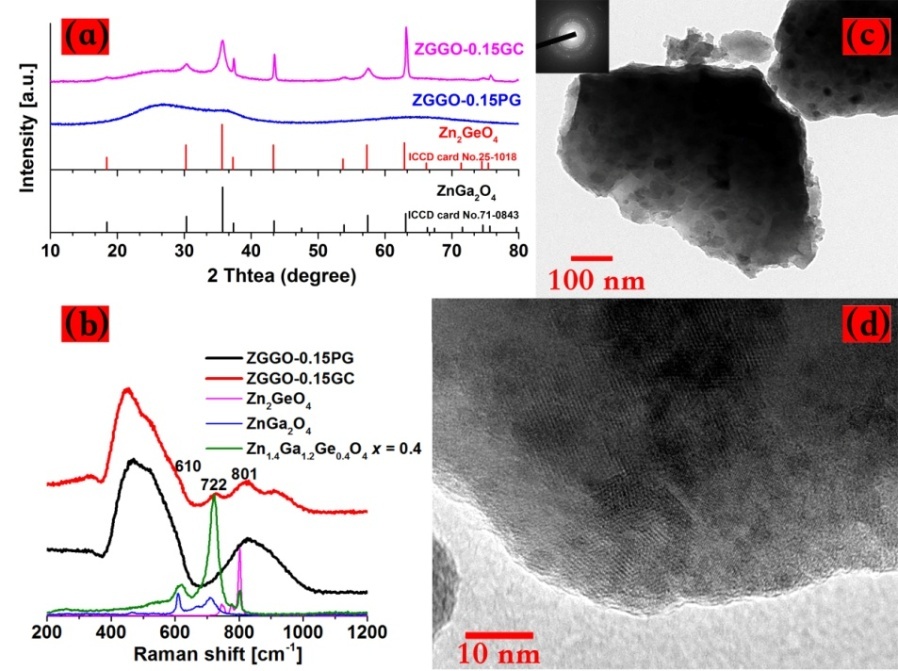


Fig. S1 (a) XRD patterns of the as-made Ni^2+^ doped glass (ZGGO-0.15PG), crystallized glass (ZGGO-0.15GC), standard ZnGa_2_O_4_ (PDF card no. 71-0843) and Zn_2_GeO_4_ (PDF card no. 25-1018) crystals; (b) Raman spectra of the as-made glass (ZGGO-0.15PG), crystallized glass (ZGGO-0.15GC), standard ZnGa_2_O_4_, Zn_2_GeO_4_ and Zn_1.4_Ga_1.2_Ge_0.4_O_4_ (*x* = 0.4) polycrystals; (c) TEM bright-field image of the crystallized glass; (d) HRTEM image of the selected area. Inset in (c): Selected-area electron diffraction (SAED) pattern.

From the XRD and Raman spectra of the ZLGO GCs, we can conclude that the ZLGO GCs contain mostly Li_2_Ge_4_O_9_ phase with only a small fraction of Zn_2_GeO_4_. For the detailed assignment of the Raman scattering peaks corresponding to Li_2_Ge_4_O_9_, refer to [[[2]](#endnote-3)].


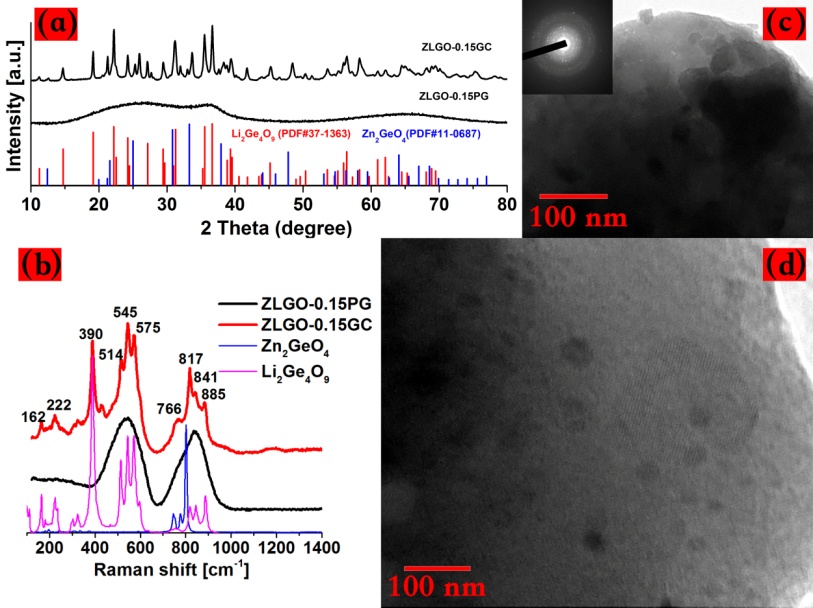


Fig. S2 (a) XRD patterns of the as-made Ni^2+^ doped glass (ZLGO-0.15PG), crystallized glass (ZLGO-0.15GC), standard Zn_2_GeO_4_ (PDF no. 11-0687) and Li_2_Ge_4_O_9_ (PDF card no. 37-1363) crystals; (b) Raman spectra of the as-made glass (ZLGO-0.15PG), crystallized glass (ZLGO-0.15GC), standard Zn_2_GeO_4_ and Li_2_Ge_4_O_9_ polycrystals; (c) TEM bright-field image of the crystallized glass; (d) HRTEM image of the selected area. Inset in (c): Selected-area electron diffraction (SAED) pattern.


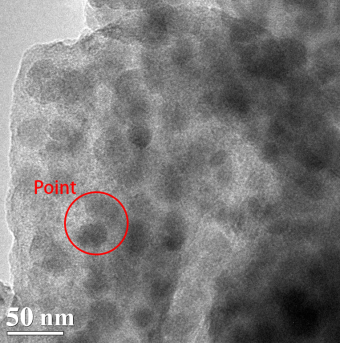

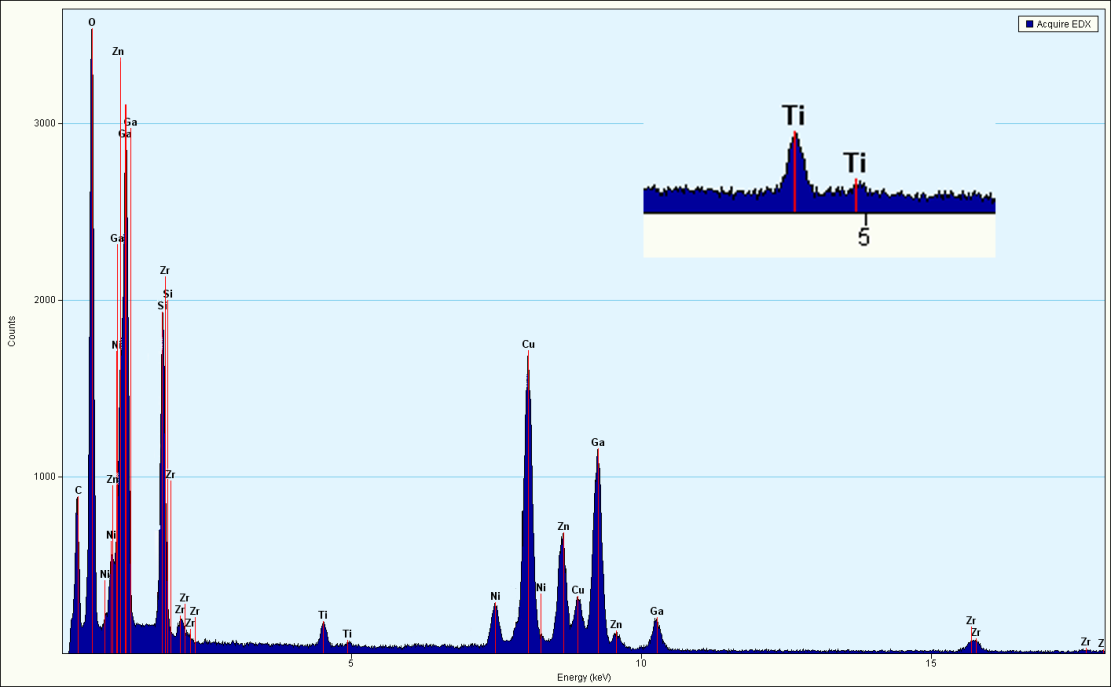


Fig. S3 TEM-EDS spectrum of the selected crystallization area (red circle in the inset TEM image) in the ZGO-0.15GC sample. The weak peaks at 4.51 and 4.93 keV are evidence of the presence of Ti in the nanoparticles. The presence of Zn and Ga elements was also recorded in the EDS spectrum, however, due to the limited resolution power of our TEM equipment (~ 0.5 mol.%), the presence of Ni was hardly detectable.

The fabricated ZGGO GCs with transmission larger than 80% demonstrate great potential to be drawn into fibers for use as fiber lasers and amplifiers.


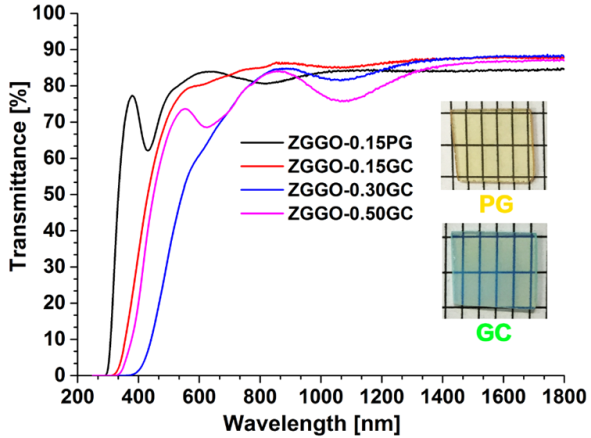


Fig. S4 Transmission spectra of the ZGGO glass and GCs of varying NiO.


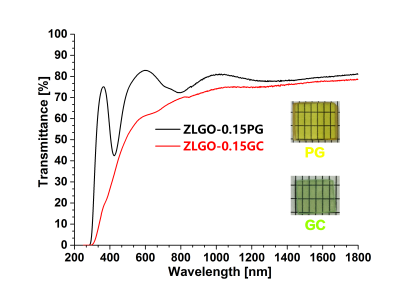


Fig. S5 Transmission spectra of the ZLGO glass (ZLGO-0.15PG) and Zn_2_GeO_4_/Li_2_Ge_4_O_9_ : Ni^2+^ GCs (ZLGO-0.15GC).

The absorption bands related to Ni^2+^ after subtracting the background absorption of glasses and GCs are shown in Fig. S5. For the ZGO and ZGGO glasses, the absorption bands can be ascribed to five-fold and four-fold coordinated Ni^2+^ as follows [[[3]](#endnote-4), [[4]](#endnote-5)]: the absorption bands around 430 nm (23256 cm^-1^), 870 (11494 cm^-1^) and 1025 nm (9756 cm^-1^) correspond to the electronic transitions from the ground state ^3^*E*'(*F*) to the excited states ^3^*A'*_2_ (*P*), ^3^*A'*_2_ (F) and ^3^*A''*_2_ (F) of the five-fold coordinated ^[5]^Ni^2+^. The gauss-fitted absorption bands around 661 (15129 cm^-1^), 715 (13986 cm^-1^) and 815 nm (12270 cm^-1^) are related to the electronic transitions from the ground state ^3^*T*_1_(*F*) to the excited states ^3^*T*_1_(*P*), ^1^*E*(*D*) and ^1^*T*_2_(*D*) of the tetrahedrally coordinated ^[4]^Ni^2+^. The profile of the absorption bands change abruptly for the crystallized glasses: two bands at 1050 nm (9500 cm^-1^) and 640 nm (15600 cm^-1^) with a shoulder at 780 nm (12900 cm^-1^) appear in the ZnGa_2_O_4_ : Ni and Zn_1+_*_x_*Ga_2-2_*_x_*Ge*_x_*O_4_ : Ni^2+^ GCs, which are typical for the octahedrally coordinated ^[6]^Ni^2+^ [[[5]](#endnote-6)]. The former two are associated with spin-allowed transitions from the ground-state ^3^*A*_2_(^3^F) to the excited ones ^3^*T*_2_(^3^F) and ^3^*T*_1_(^3^F), and that at 780 nm stems from the spin-forbidden transition to ^1^*E*(^1^*D*) state [Error: Reference source not found]. Differences can be noticed from ZLGO glasses as compared to ZGO and ZGGO glasses, viz., the absorption bands due to ^[6]^Ni^2+^ also exists in addition to those of ^[5]^Ni^2+^ and ^[4]^Ni^2+^. As for the ZLGO GCs, ^[4]^Ni^2+^ coexists with ^[6]^Ni^2+^, indicating an incomplete transformation from ^[5]^Ni^2+^ and ^[4]^Ni^2+^ to ^[6]^Ni^2+^ upon crystallization. The assignment of the absorption bands in the present investigation refers to some of the most cited papers [Error: Reference source not found, Error: Reference source not found, [[6]](#endnote-7)].

It should be stressed that the unambiguous assignment of the absorption bands to Ni^2+^ of specific coordination states in glasses and GCs remains to a challenging task because of the availability of various coordination environments for Ni^2+^ ions: they can reside in the residual glass (with a different environment compared to the initial glass due to changes in the composition of the remaining glassy part), in one or several different crystalline sites, and at the glass/crystal interfaces [[[7]](#endnote-8)].


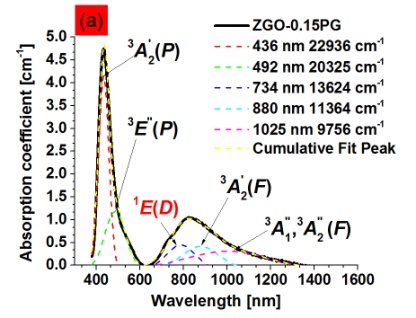

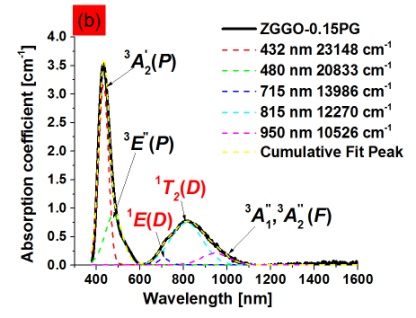

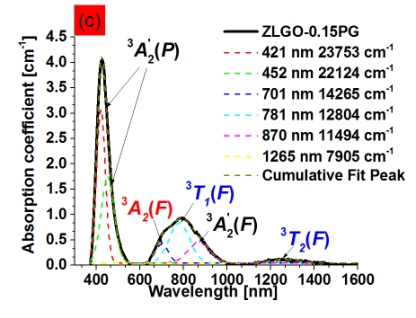


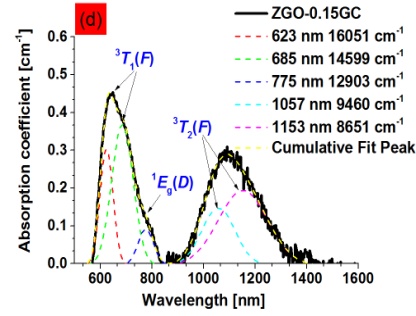

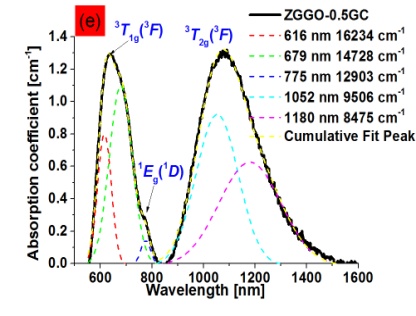

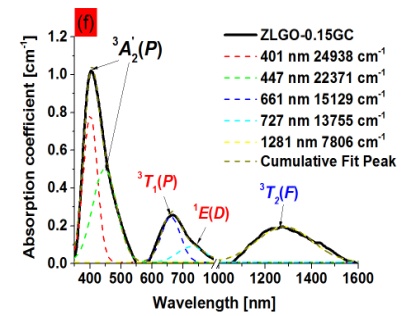


Fig. S6 Absorption spectra obtained after subtracting the background absorption of glasses and GCs. The absorption bands are fitted by Gauss function. The absorption bands related to ^[6]^Ni, ^[5]^Ni, ^[4]^Ni are indicated by blue, black and red color, respectively.


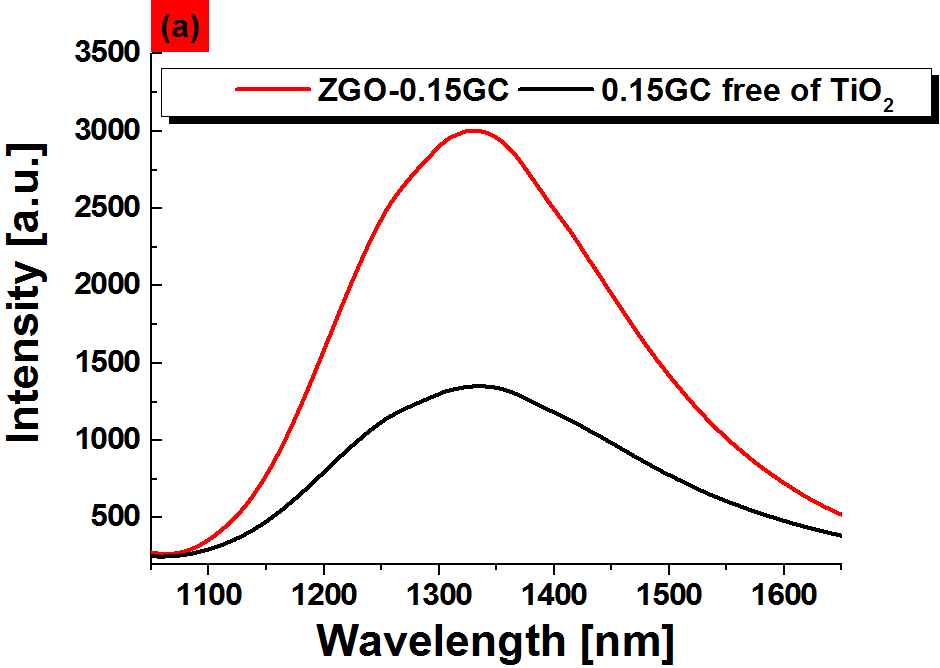

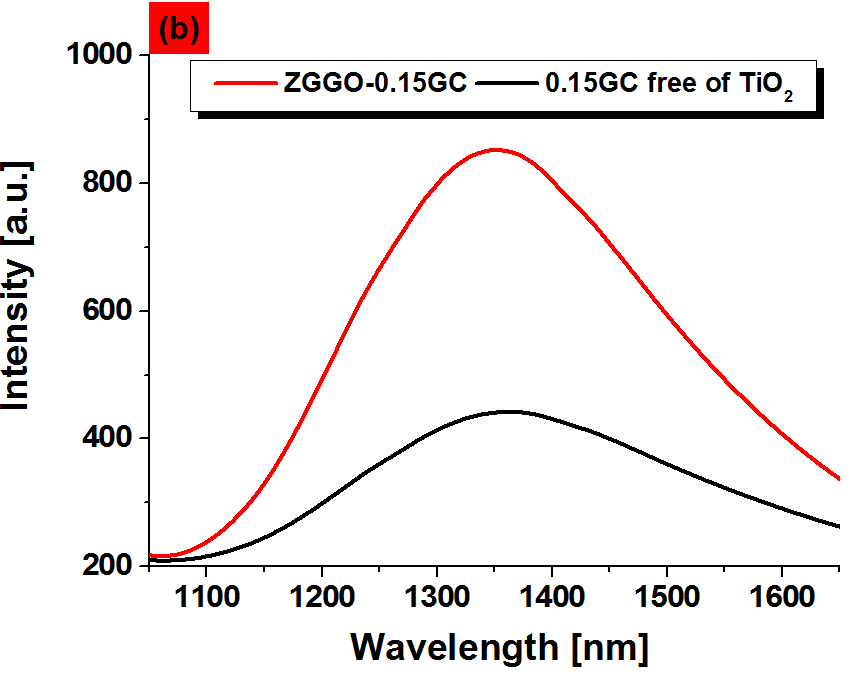


Fig. S7 (a) Emission spectra of the ZGO GCs containing (red line) and free of TiO_2_ (black line), and (b) ZGGO GCs containing (solid lines) and free of TiO_2_ (dashed lines), respectively. Here only the 0.15 mol.% NiO doped GC samples were shown as an example.


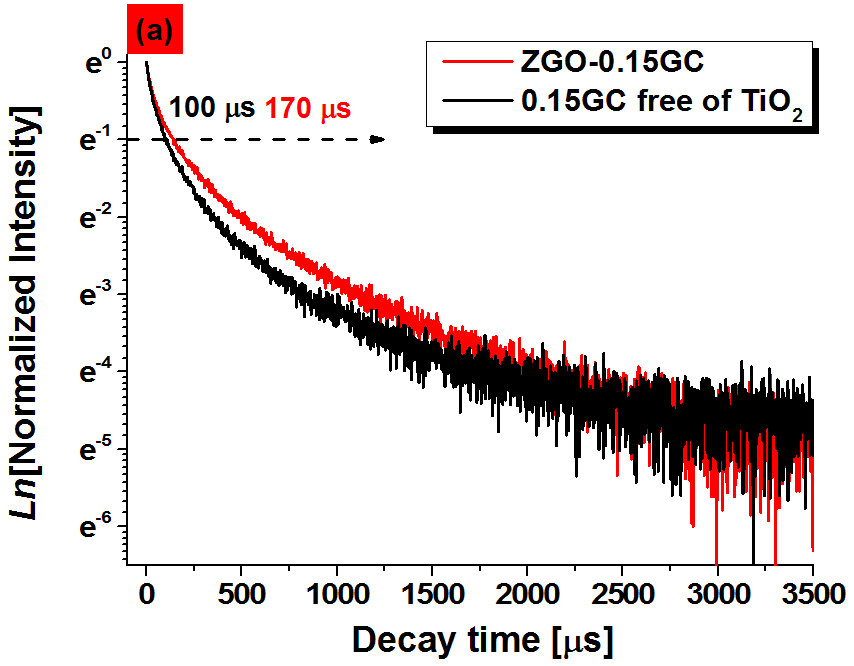

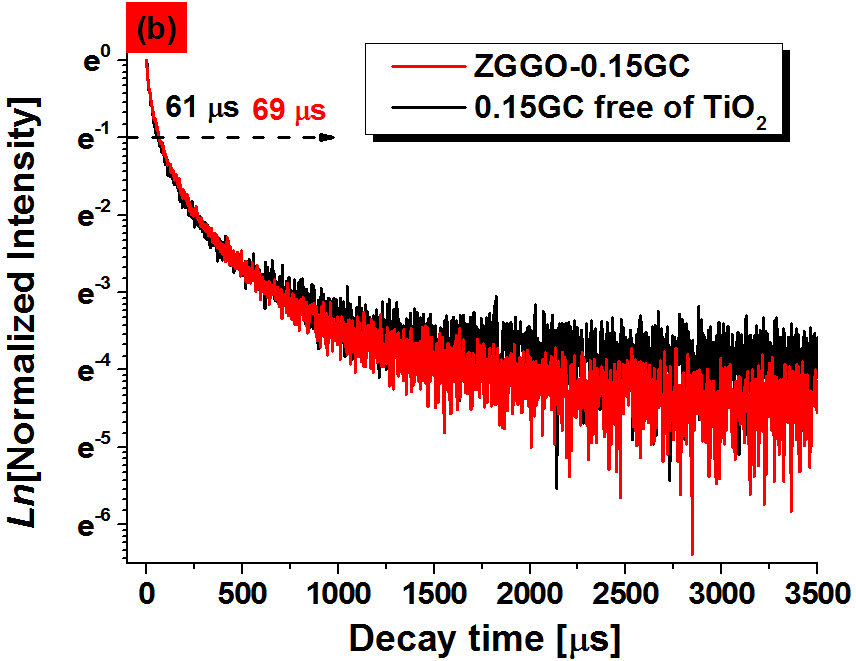


Fig. S8 (a) Decay curves of the ZGO GCs containing (red line) and free of TiO_2_ (black line), and (b) ZGGO GCs containing (red line) and free of TiO_2_ (black line), respectively. Here only the 0.15 mol.% NiO doped GC samples were shown as an example.


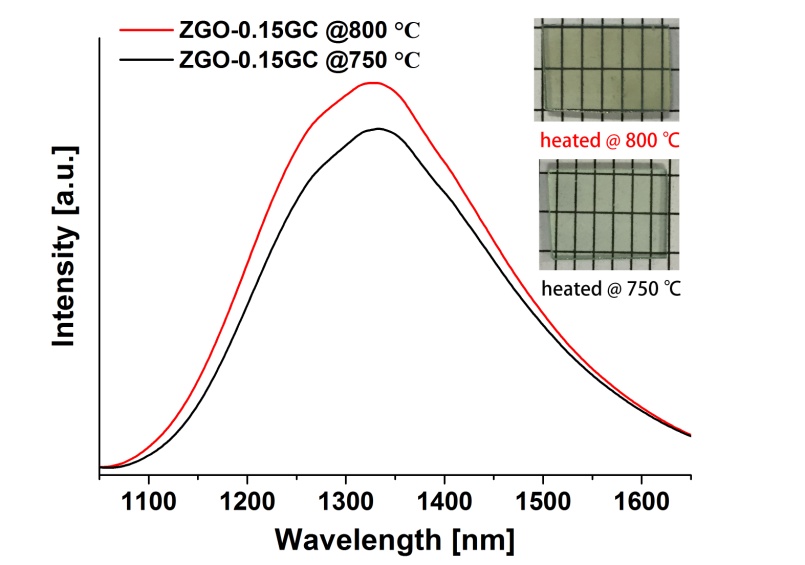


Fig. S9 Emission spectra of the ZGO-0.15GC samples obtained by thermal treatment at 750 and 800 ^o^C for 10 hours, respectively. Inset: photos of the GC samples showing excellent transparency.

**References**

1. J. Ren, X. Xu, H. Zeng, G. Chen, D. Kong, C. Gu, C. Chen, Z. Liu and L. Kong, Novel self-activated zinc gallogermanate phosphor: The origin of its photoluminescence, *J. Am. Ceram. Soc.* **97,** 3197-3201 (2014) [↑](#endnote-ref-2)
2. V. N. Sigaev, S. V. Lotarev, E. N. Smelyanskaya, P. D. Sarkisov, A. A. Volkov, G. A. Komandin, V. V. Koltashev, and V. G. Plotnichenko, Raman and Dielectric Spectra of the Glass and Single Crystal of the Composition Li_2_Ge_7_O_15_ in the Frequency Range 3-1000 cm^-1^: II. The Influence of Phase Separation, Glass Physics and Chemistry, 32[5], 497-504 (2006) [↑](#endnote-ref-3)
3. L. Galoisy, and G. Calas, Structural environment of nickel in silicate glass/melt systems: Part 1. Spectroscopic determination of coordination states, Geochim. Cosmochim. Acta 57, 3613-3626 (1993) [↑](#endnote-ref-4)
4. L. Galoisy, G. Calas, L. Cormier, B. Marcq, M. H. Thibault, Overview of the environment of Ni in oxide glasses in relation to the glass colouration, Phys. Chem. Glasses. 46[4], 394-399 (2005) [↑](#endnote-ref-5)
5. T. Suzuki, G. S. Murugan, Y. Ohishi, Spectroscopic properties of a novel near-infrared tunable laser material Ni : MgGa_2_O_4_, J. Lumin. 113, 265-270 (2005) [↑](#endnote-ref-6)
6. A. Dugue, L. Cormier, O. Dargaud, L. Galoisy, and G. Calas, Evolution of the Ni^2+^ Environment During the Formation of a MgO-Al_2_O_3_-SiO_2_ Glass-Ceramic: A Combined XRD and Diffuse Reflectance Spectroscopy Approach, J. Am. Ceram. Soc. 95[11], 3483-3489 (2012) [↑](#endnote-ref-7)
7. A. Dugué, O. Dymshits, L. Cormier, B. Cochain, G. Lelong, S. Belinc, A. Zhilin, Structural evolution of Ni environment in lithium, magnesium and zinc aluminosilicate glasses and glass-ceramics, J. Non-Crystal. Solids 413, 24-33 (2015) [↑](#endnote-ref-8)
